# Dietary miR-451 protects erythroid cells from oxidative stress via increasing the activity of Foxo3 pathway

**DOI:** 10.18632/oncotarget.22346

**Published:** 2017-11-10

**Authors:** Wanchen Wang, Chengwen Hang, Yanqing Zhang, Mingshi Chen, Xinyu Meng, Qing Cao, Nana Song, Jacobi Itkow, Feiyang Shen, Duonan Yu

**Affiliations:** ^1^ Jiangsu Key Laboratory of Experimental & Translational Non-coding RNA Research, Yangzhou University School of Medicine, Yangzhou 225001, China; ^2^ Institute of Comparative Medicine, Yangzhou University, Yangzhou 225001, China; ^3^ Institute of Translational Medicine, Yangzhou University School of Medicine, Yangzhou 225001, China; ^4^ Jiangsu Co-Innovation Center for Prevention and Control of Important Animal Infectious Disease and Zoonosis, Yangzhou 225001, China

**Keywords:** microRNA, food safety, *miR-144/451* knockout mice, erythroid cell

## Abstract

One fundamental issue in public health is the safety of food products derived from plants and animals. A recent study raised a concern that microRNAs, which widely exist in everyday foods, may alter consumers’ functions. However, some studies have strongly questioned the likelihood of dietary uptake of functional microRNAs in mammals. Here we use a microRNA gene knockout animal model to show that *miR-144/451* null mice can orally uptake miR-451 from a daily chow diet, and ingestion of wild type blood, that contains abundant miR-451, also enhances the level of miR-451 in the circulating blood of knockout mice. Moreover, reducing miR-451 level in *miR-144/451* knockout blood by consuming food lacking miR-451 reduces the anti-oxidant capacity of *miR-144/451* null red blood cells by targeting the 14-3-3ζ/Foxo3 pathway, while increasing miR-451 level via gavage-feeding of wild type blood increases the anti-oxidant capacity of *miR-144/451* null red blood cells. We conclude that 1) some miRNAs in food can pass through the gastrointestinal tract into the blood to affect consumers’ function and 2) microRNA knockout animals such as *miR-144/451* null mice can acquire the deleted genetic information from daily foods, which might alter the results and conclusions from the studies using such animals.

## INTRODUCTION

Systemic exposure to dietary RNAs from consumption of plant or animal products is considered limited in high organisms, presumably by extensive degradation of RNAs in the gastrointestinal (GI) tract and the rapid catabolism of RNAs in cells [1]. Preclinical and clinical data on systemic delivery of oligonucleotide therapeutics also indicate limited cellular uptake of RNAs. However, a recent study published in *Cell Research* reported a striking finding that orally acquired microRNAs (miRNAs), a class of small RNA whose length is 18-25 base pairs (bp), from rice were efficiently detected in mammalian circulating blood and tissues by sequencing and quantitative PCR. Furthermore, these plant miRNAs were found to be able to regulate mammalian gene expression [2]. This discovery of so-called cross-kingdom regulation of gene expression was confirmed or partially confirmed by other laboratories or in other experimental settings [3-12], but strongly questioned by some authors in light of their negative results [13-18]. Clearly, controversy exists for whether exogenous RNA, especially miRNAs, can be absorbed by the digestive system to affect the function of a living organism. Scientific issues but also technical issues may be the underlining causes of inconsistency [19]. Contamination of dietary miRNAs with the endogenous mammalian miRNAs may also lead to the false positive results.

Here we use a simple animal model, *miR-144/451* gene knockout (KO) mice [20], to clearly demonstrate that ingestion of wild type (WT) blood, that contains abundant miR-451 and miR-144, significantly increases the level of miR-451 and miR-144 in the circulation of *miR-144/451* mutant mice. In addition, miR-451 can be sufficiently detected in the circulating blood of *miR-144/451* KO mice fed with regular mouse chow diet. We further demonstrate that these dietary miR-451 molecules existing in the blood stream, even at very low levels, are capable of inhibiting their target gene expression in animals. Our study supports the finding that miRNAs, which exist as active ingredients in some everyday foods or dietary supplements, can affect the functions of the consumer. Our finding also raises concerns about the acquisition of the deleted miRNAs from regular chow diet, which might skew the results from the studies using miRNA KO animals.

## RESULTS

### Ingestion of wild type blood increases the levels of miR-451 and miR-144 in peripheral blood of *miR-144/451* null mice

**Figure 1:**
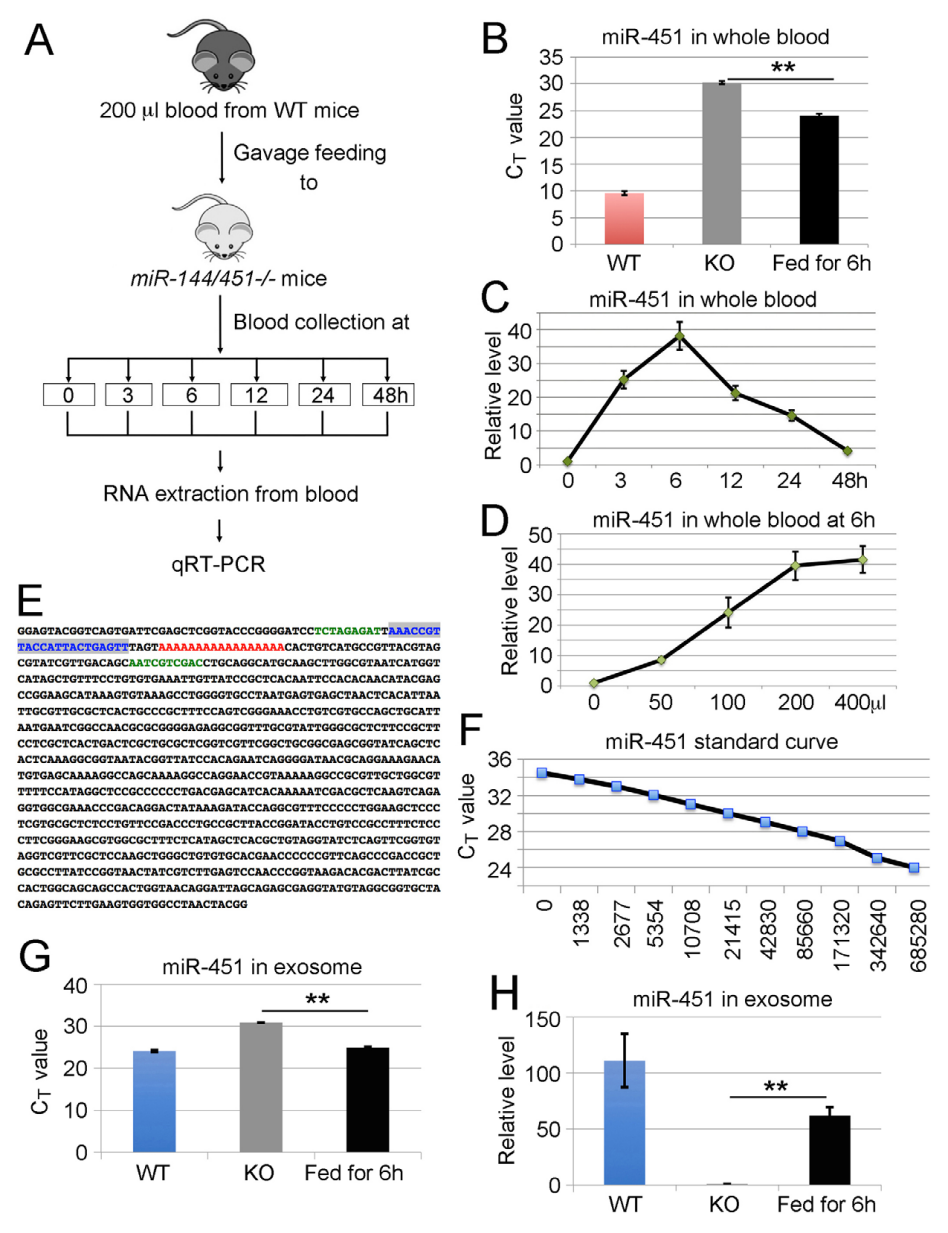
Ingestion of wild type blood increases the levels of miR-451 and miR-144 in peripheral blood of *miR-144/451* null mice **(A)** Schematic view for the ingestion of WT blood into *miR-144/451* KO mice. *miR-144/451* KO mice were gavage fed with 200 μl fresh blood from WT animals. Blood was then drawn from the KO mice at different time points ranging from 0 to 48 hours. 200 μl of whole blood was used for RNA extraction and miR-451 levels in blood were assessed by two different PCR methods: TaqMan probe-based qRT-PCR assay and All-in-One miRNA detection kit. **(B)** Quantitative analysis of miR-451 levels in peripheral blood of *miR-144/451* KO mice 6 hours after feeding WT blood. The Y-axis shows the C_T_ value of miR-451. WT blood was used as positive control. **(C)** Quantitative analysis of miR-451 levels in peripheral blood of *miR-144/451* KO mice after ingestion of WT blood. The Y-axis shows relative fold change of miR-451 with the miR-451 level at zero hour assigned as a relative value of 1. X-axis shows hours after feeding WT blood. Data is from three independent experiments. **(D)** qRT-PCR analysis of miR-451 expression 6 hours after gavage feeding different amounts of WT blood. The Y-axis shows relative levels of miR-451 in *miR-144/451* KO blood. X-axis shows amount of WT blood fed to *miR-144/451* KO mice. Data is from three independent experiments. **(E)** Sequence of PCR product. PCR products were gel-purified, cloned and sequenced. Note: the sequence highlighted with blue color is the sequence of mature miR-451 and the poly A highlighted with red color is the adapter sequence for reverse transcription. **(F)** Standard curve for miR-451. C_T_ values were plotted against copy number. **(G-H)** Quantitative analysis of miR-451 levels in exosomes isolated from peripheral blood of *miR-144/451* KO mice 6 hours after ingestion of WT blood. The Y-axis shows the C_T_ value of miR-451 (G) and fold change of miR-451 with the miR-451 level at zero hour (KO) assigned as a relative value of 1 (H). WT blood was used as positive control. The loading control for all miRNA detection was small nuclear RNA U6.

Our previous study shows that miR-451 and miR-144 are encoded by a bicistronic miRNA locus transcriptionally controlled by GATA1, a “master” nuclear factor in erythroid cells [21]. The strong elevation of miR-144/451 during erythroid differentiation makes miR-144/451 the most abundant miRNAs in mature red blood cells. Specifically, miR-451 comprises about 50% of the total miRNA pool in murine erythrocytes as reported by an RNA sequencing study [22]. To investigate whether miR-144/451 can pass through various barriers in the digestive system into circulating blood, *miR-144/451* KO mice [20] were gavage fed with different amounts of fresh blood from WT animals. Blood was then drawn from KO mice at different time points ranging from 0 to 48 hours following the uptake of the WT blood. 200 μl of whole blood was used for RNA extraction and miR-144/451 levels were assessed by two different PCR methods: TaqMan probe-based quantitative real time PCR (qRT-PCR) assay and All-in-One miRNA detection kit. Both qRT-PCR methods, using small nuclear RNA U6 (snRU6, or U6) as internal loading control, showed that the miR-451 level in peripheral blood of *miR-144/451* KO mice rapidly climbed after feeding WT blood and reached a peak after 6 hours (Figure 1A-1C). The degree of the miR-451 increase in *miR-144/451* KO blood was dependent on the amount of blood orally received by the *miR-144/451* KO mice, and administrating 200-400 μl of blood gave the maximum increase of miR-451 in blood (Figure 1D). Interestingly, feeding synthetic miR-451 to *miR-144/451* KO mice also increased the miR-451 level in *miR-144/451* KO blood, but the degree of which miR-451 increased was far less than when fed with WT blood (Supplementary Figure 1A-1B). To examine whether the PCR signals are from miR-451, PCR products from *miR-144/451* KO blood, either before or after gavage feeding of WT blood, were gel-purified, engineered to TA cloning vector and sequenced. As expected, the sequence for mature miR-451 (blue color) followed by a poly-A sequence (a link sequence for reverse transcription, red color) existed in PCR products (Figure 1E), confirming that the mature miR-451 molecules were in the circulation of *miR-144/451* KO mice. To estimate the copy numbers of mature miR-451 that existed in *miR-144/451* KO blood, a standard curve was made using synthetic miR-451 as input ranging from 0.015625 pg/μl (equivalent to 1338 copies) to 4 pg/μl (equivalent to 685280 copies, http://www.endmemo.com/bio/dnacopynum.php) per PCR reaction (Figure 1F). Since the average threshold cycle (C_T_) per PCR reaction for *miR-144/451* KO blood sample was around 30, we estimated that the copy number of miR-451 in *miR-144/451* KO blood before and after gavage feeding of WT blood were 1.07 x 10^5^ copies/μl and 4.28 x 10^6^ copies/μl blood, respectively. The level of miR-144 in *miR-144/451* KO peripheral blood also climbed after ingestion of WT blood (Supplementary Figure 1C-1D), although miR-144 as well as miR-451 levels were lower by an order of magnitude when compared to those in WT blood (Figure 1B, Supplementary Figure 1C), and the majority of the dietary miR-144/451 were presumably destroyed or blocked by the digestive system.

To investigate whether miR-451 exists in exosomes, exosomes were collected from *miR-144/451* KO peripheral blood before and after gavage feeding of WT blood. The purified exosomes were then used for RNA extraction. Exosomes purified from WT blood were used as control. Consistent with our previous finding, the C_T_ value for *miR-144/451* KO exosomes without gavage feeding of WT blood was around 30 (Figure 1G). After feeding with WT blood, the C_T_ was down about 6 cycles, meaning miR-451 increased 60 fold (Figure 1G–1H). The level of miR-451 in serum depleted of exosomes with ultracentrifugation (120,000g for 2 hours) was in undetectable range (data not shown). This data suggests that exosomes may be the transportation vesicle for miR-144/451 transferring from the GI tract into peripheral blood, or at least, exosomes are used as the transportation vesicles by miR-451 molecules when they are circulating in vascular system.

**Figure 2:**
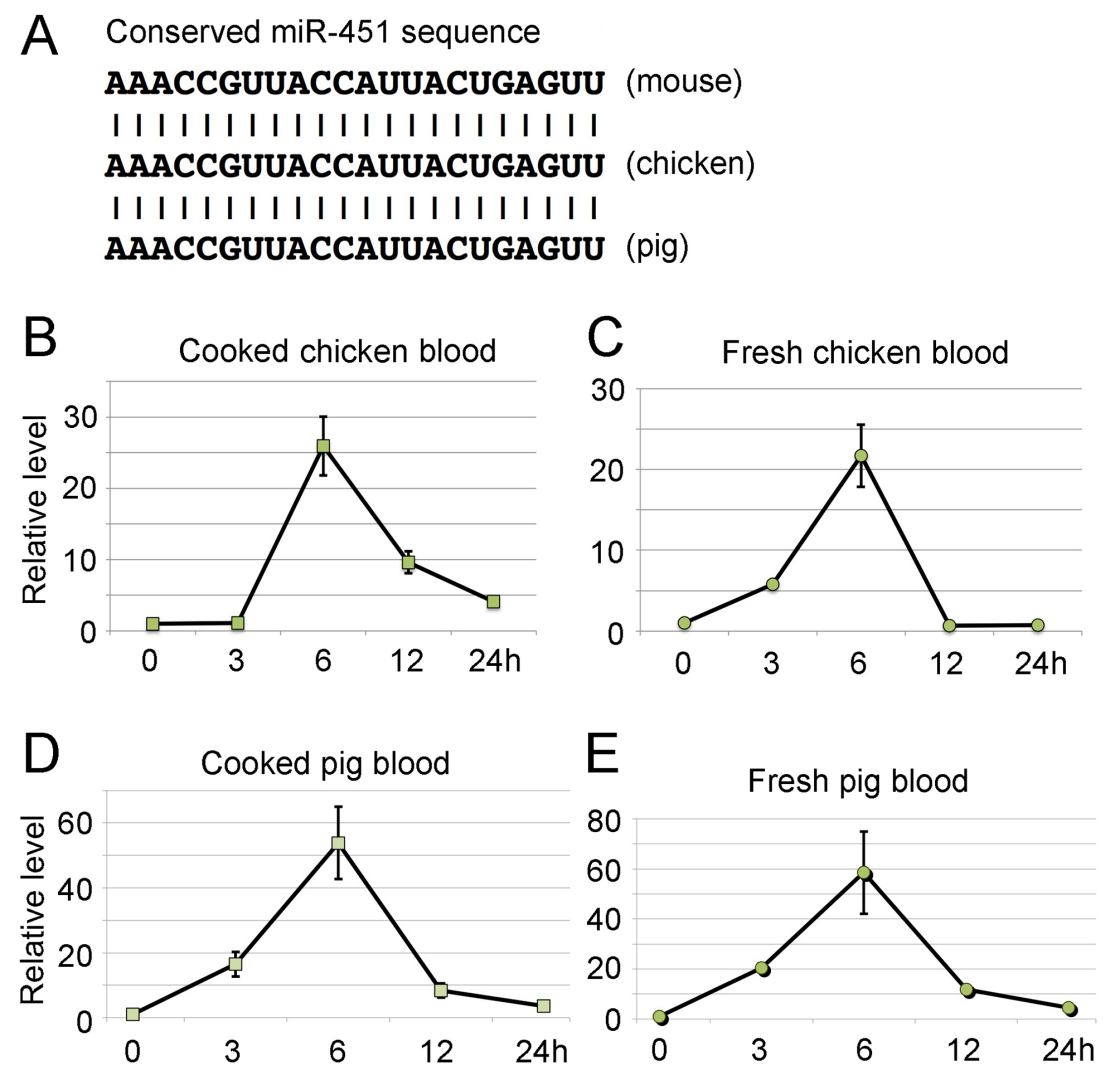
Ingestion of wild type blood from chickens and pigs increases the level of miR-451 in peripheral blood of *miR-144/451* KO mice **(A)** Multispecies nucleotide sequence alignments showing complete conservation of mature miR-451 in mouse, chicken and pig. Quantitative PCR analysis of miR-451 expression after gavage-feeding cooked chicken blood **(B)** fresh chicken blood **(C)** cooked pig blood **(D)** and fresh pig blood **(E)**. The Y-axis shows relative level of miR-451 in *miR-144/451* KO blood. X-axis shows time (hour) after feeding various types of blood. Represented data is from five different mice.

miR-144/451 is highly conserved in different species ranging from human to zebra fish [21]. The sequences of mature miR-451 are 100% identical among mice, chickens and pigs (Figure 2A). To examine whether miR-451 from chickens and pigs, two animals most common in human diets, can go through the mouse digestive system, blood from chicken and pig, either fresh or cooked, were gavage fed to *miR-144/451* KO mice. Consistent to the results from administration of WT mouse blood, the level of miR-451 was significantly elevated after ingestion of chicken or pig blood (Figure 2B-2E). These results clearly indicate that exogenous miR-451 can enter the circulation through the mouse digestive system.

### Ingestion of WT splenocytes slightly increases miR-15a level in *miR-15a/16-1* KO blood but fails to increase *lnk* mRNA in *lnk* KO mouse blood

**Figure 3:**
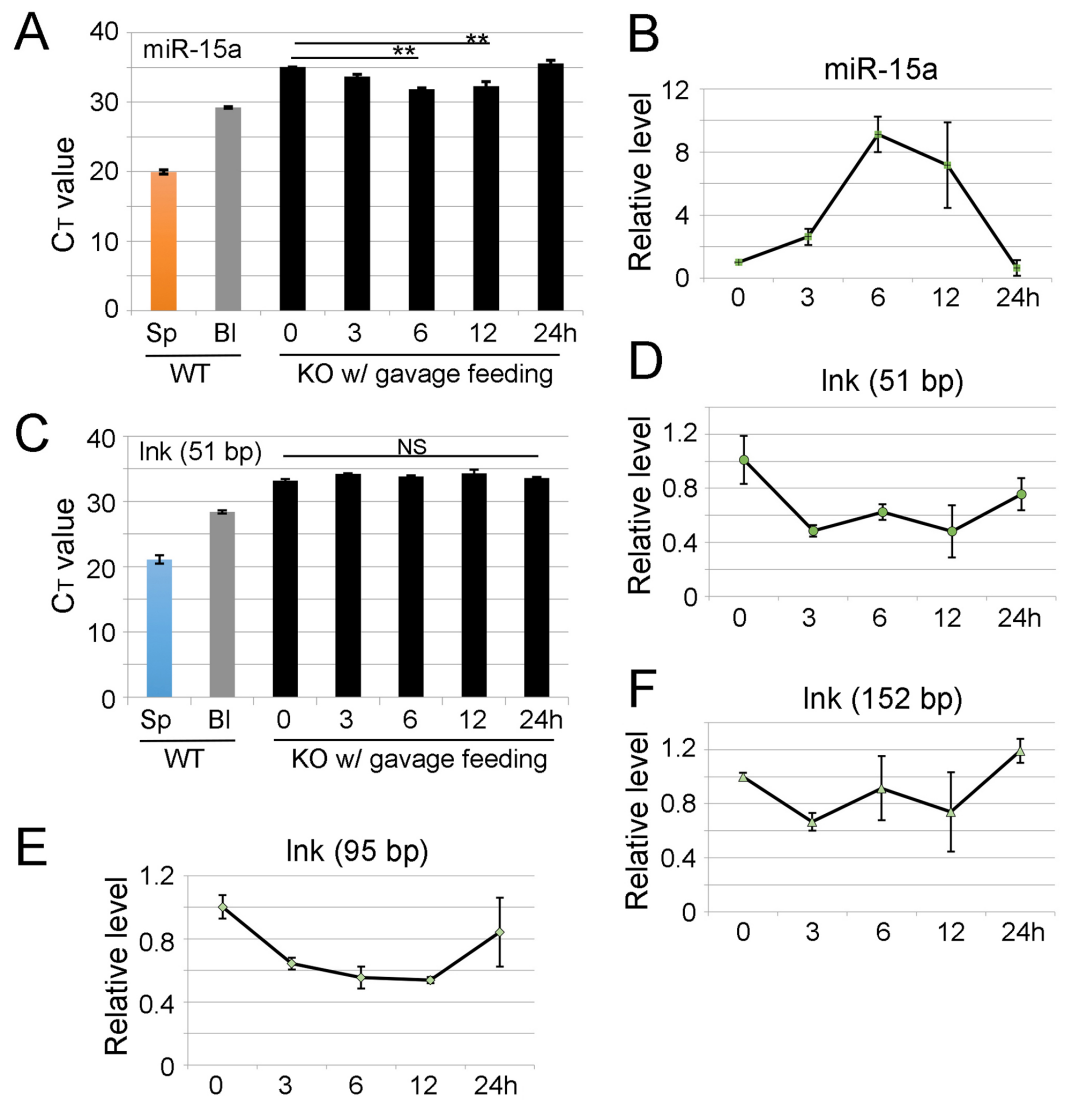
Ingestion of wild type splenocytes slightly increases miR-15a level in *miR-15a/16-1* KO blood but fails to increase *lnk* mRNA in *lnk* KO mouse blood Each *miR-15a/16-1* KO mouse was gavage fed with 20 million fresh splenocytes in 200 μl suspension from WT animals. Blood was then drawn from the KO mice at different time points following uptake of the WT splenocytes. miR-15a level in blood was assessed by qRT-PCR. **(A)** Quantitative analysis of different miR-15a levels in *miR-15a/16-1* KO blood, the Y-axis shows C_T_ value. **(B)** Y-axis shows relative level of miR-15a in *miR-15a/16-1* KO blood. snRU6 was the internal control. **(C)** Quantitative analysis of different *lnk* mRNA levels in peripheral blood of *lnk* KO mice after ingestion of same amount of WT splenocytes. The Y-axis shows C_T_ value. **(D-F)** Y-axis shows relative fold change of *lnk* mRNA fragments. X-axis shows hours after feeding WT splenocytes. Mouse *beta-actin* mRNA was used as the loading control. Data is from three independent experiments. Note: there are no fragments longer than 50 bp increased in *lnk* KO blood after gavage feeding with WT splenocytes and the high C_T_ value may reflect the background noise.

To examine whether the mouse digestive system allows the increase of other dietary miRNAs in circulating blood, 20 million splenocytes from WT mice were used to orally feed each *miR-15a/16-1* KO mouse. miRNAs encoded by the *miR-15a/16-1* locus (*miR-15a* and *miR-16-1*) function as tumor suppressors and are abundantly expressed in white blood cells, especially in lymphocytes [23]. Consistent with the results from miR-144/451 experiments, mature miR-15a was detected in recipient *miR-15a/16-1* KO mouse blood after ingestion of WT splenocytes, but the degree of increase was much lower compared with that of miR-451 in *miR-144/451* KO blood (Figure 3A-3B).

To unravel whether RNA from protein coding genes can pass through the GI tract, splenocytes from WT mice were orally administrated to *lnk* gene KO mice [24]. Lnk is a SH2 domain-containing adaptor protein expressed preferentially in lymphocytes. The full-length *lnk* cDNA has 1888 bps. Considering the faster degradation of long RNA molecules, qRT-PCR primers were designed for detection of different sized *lnk* mRNA fragments (51 bp, 95 bp and 152 bp). There was no increase of any *lnk* RNA fragments longer than 50 bps in *lnk* KO blood following the oral uptake of WT splenocytes (Figure 3C-3F), suggesting sufficient degradation or blocked absorption of *lnk* mRNA. The low C_T_ value (around 35 cycle, Figure 3C) of *lnk* qRT-PCR presumably reflects the undetectable range or the background signals.

### Ribonuclease degrades miR-451 efficiently *in vitro*

**Figure 4:**
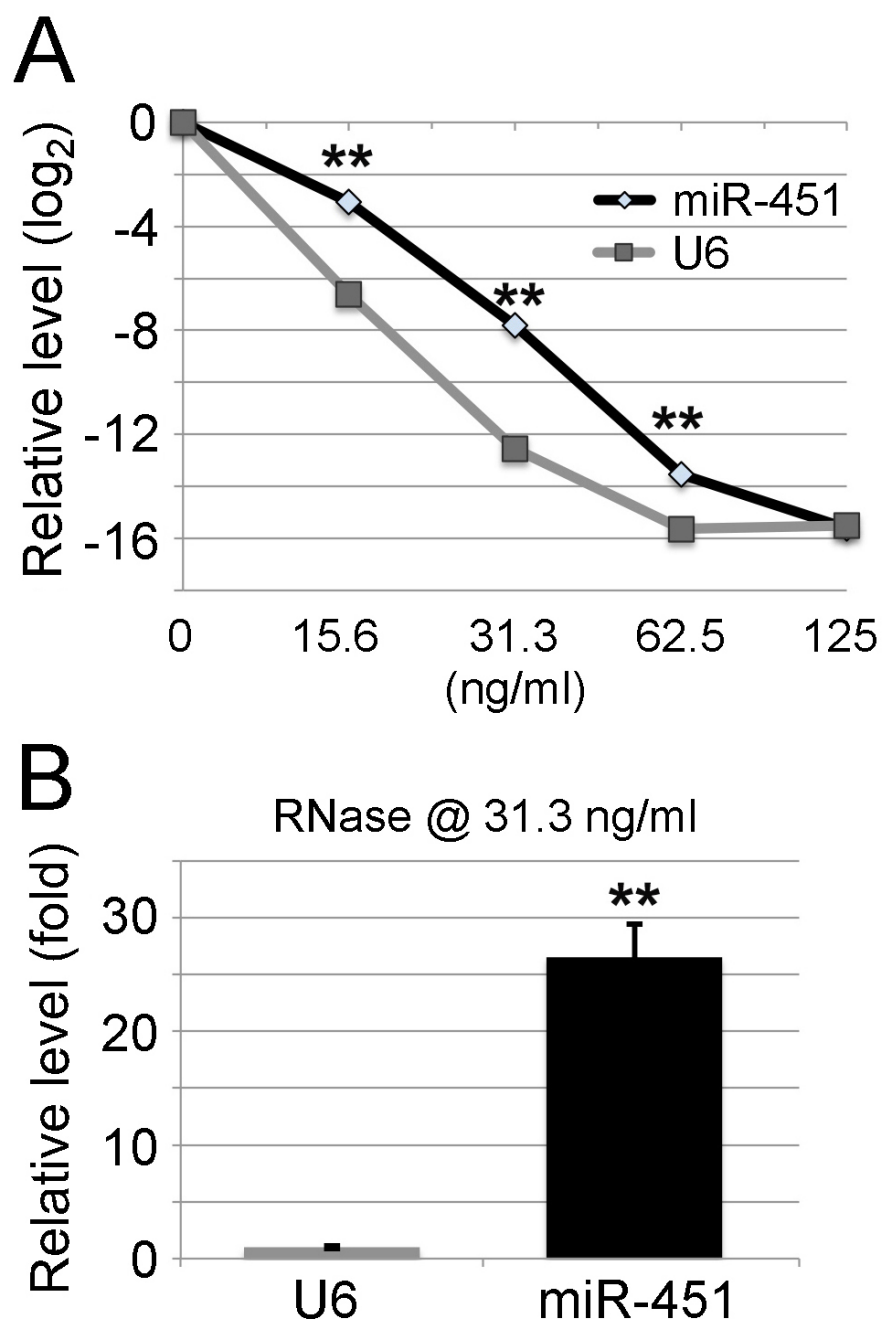
RNase degrades miR-451 efficiently *in vitro* Total RNA from WT blood was treated with RNase A at different concentrations for 30 min at room temperature. **(A)** miR-451 level after RNase treatment. Relative expression levels are shown on the Y-axis with RNase untreated samples assigned a relative value of 1 (0 on log2 scale). X-axis shows the concentrations of RNase A. As expected, RNase treatment significantly affects the stability of miR-451 in a dose dependent manner. However, miR-451 has a slower degradation rate compared with small nuclear RNA U6 after RNase treatment. Data represents 3 different RNA samples. **(B)** The fold difference between miR-451 and U6 levels after the treatment with RNase at the concentration of 31.3 ng/ml as shown in (A). Note: miR-451 survives degradation by RNase better than U6.

To investigate why the majority of miR-144/451 in orally ingested WT red blood cells cannot reach the circulating blood of recipient *miR-144/451* KO mice, a series of experiments were performed to examine whether ribonuclease (RNase), high temperature, acidification, or alkaline treatments can degrade miR-451. To examine whether RNase destroys miR-451, total RNA from fresh WT blood was treated with different concentrations of RNase for 30 min. As expected, RNase treatment significantly affected the stability of miR-451 in a dose dependent manner and even trace amount of RNase (15.6 ng/ml) degraded miR-451 by an order of magnitude (Figure 4A). Interestingly, miR-451 had a much slower degradation rate compared with the small nuclear RNA U6 after RNase treatment (Figure 4A). RNase at the concentration of 31.3 ng/ml degraded U6 about 26 times more than miR-451 (Figure 4B). This data clearly indicates that RNase, presumably existing during food digestion, can efficiently degrade miRNAs.

To mimic food processing, an experiment was set to determine whether heating or regular cooking (95°C for 10 min) could degrade miR-451. As shown in Supplementary Figure 2, there was no significant difference of miR-451 levels between fresh and cooked blood from three different species (mouse, chicken and pig). Like in a human stomach, mouse gastric fluid is acidic but has slightly higher pH ranging from 3.0 to 4.0, depending on the amount of food the animal ingests [25]. To mimic this acidic environment, red blood cells from WT mice were incubated at 37°C for two hours with either an acidic solution whose pH was adjusted to 2.5 or a simulated gastric fluid (SGF) [26]. The acid treatment failed to significantly change the yield of miR-451 from WT mouse blood (Supplementary Figure 3A-3B). To mimic the alkaline environment of mouse blood whose pH is around 7.4, as well as the possible blood alkaline tide that usually occurs after food digestion, RNA from WT mouse blood was either adjusted with a NaOH solution to pH 8.5 or was added to simulated intestine fluid (SIF) [26] for two hours at 37°C. No significant difference of miR-451 levels between alkaline solution treated and untreated RNA was observed (Supplementary Figure 3C-3D). The data indicates that miR-451 can survive high temperature, acidification, and alkaline conditions.

### Chow diet-derived miR-451 is present in *miR-144/451* KO mice

**Figure 5:**
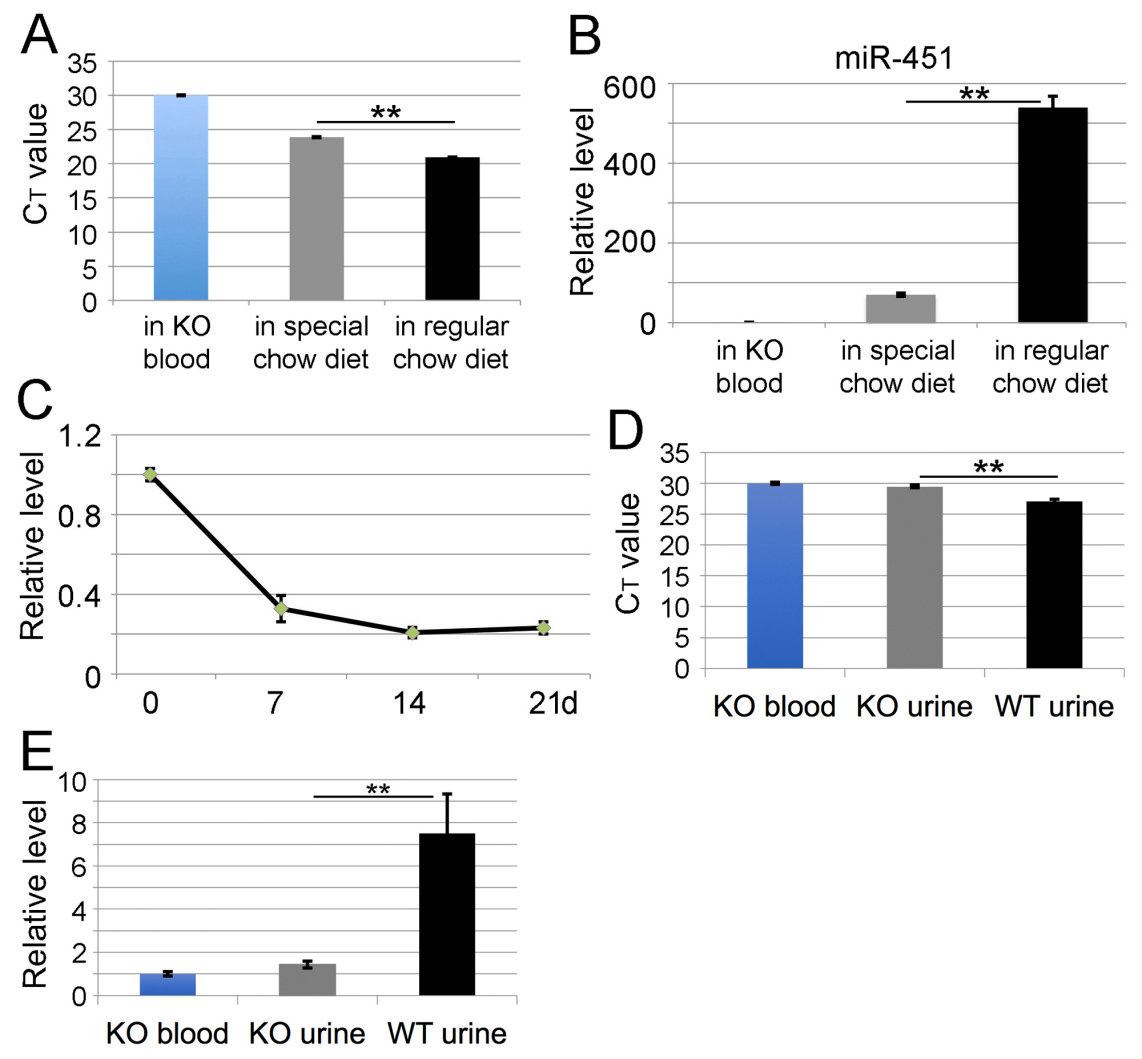
Chow diet-derived miR-451 is present in *miR-144/451* KO mice Analysis of **(A)** C_T_ value and **(B)** relative level of miR-451 in different mouse chow diet. X-axis shows two different chow diets. miR-451 level in *miR-144/451* KO blood was used as control and assigned a relative value of 1. **(C)** qRT-PCR analysis of blood miR-451 level in *miR-144/451* KO mice after feeding with a special chow diet that contains a very low level of miR-451 (B). The Y-axis shows relative level of miR-451 in *miR-144/451* KO blood. X-axis shows the time (day) when the blood was drawn after feeding *miR-144/451* KO mice with special chow diet. Data is from three independent experiments. Analysis of C_T_ value **(D)** and relative level **(E)** of miR-451 in mouse urine. miR-451 level in *miR-144/451* KO blood was used as control.

When miR-144/451 levels in blood samples from *miR-144/451* KO mice were semi-quantitatively measured with qRT-PCR, the C_T_ values were consistently around 28-32 cycles. We thus hypothesized that the miR-144/451 signals from qRT-PCRs were either background noise or real signals from regular mouse chow diet containing fish powder as supplement. Given that miR-451 is highly conserved among different species such as human, mouse and many different types of fishes including zebra fish, we hypothesized that *miR-144/451* KO mice might uptake food-derived miR-451. To test this hypothesis, the regular chow diet was first used for RNA extraction and miR-451 was measured with qRT-PCR. In agreement with the hypothesis, a considerable level of miR-451 was detected in the regular chow diet compared with the miR-451 levels in *miR-144/451* KO blood and a special chow diet containing no fish powder (Figure 5A-5B). These diet-derived miR-451 are stable for at least three weeks when the chow diet is kept at room temperature (data not shown). To examine whether the potentially existent miR-451 in *miR-144/451* KO blood was from food uptake, the *miR-144/451* KO mice originally fed with a regular chow diet were fed with the special chow diet. The level of miR-451 decreased three-fold in the first week and four-fold after two weeks (Figure 5C), indicating that the miR-451 was reduced to levels that make detection difficult. To investigate whether food-derived miR-451 can pass through the urinary system, we measured miR-451 levels in urine from WT and *miR-144/451* KO mice using *miR-144/451* KO blood as control. miR-451 levels in the urine of *miR-144/451* KO mice are similar to the ones in *miR-144/451* KO blood (C_T_ value around 30). Interestingly, there are only several folds more miR-451 in WT urine than *miR-144/451* KO urine (Figure 5D–5E), suggesting that miR-144/451 undergo catabolism before clearance by urinary system. These results confirm that *miR-144/451* KO mice still contain miR-451 primarily coming from the mouse chow diet.

### Dietary miR-451 protects against oxidant stress in *miR-144/451* KO erythroid cells

**Figure 6:**
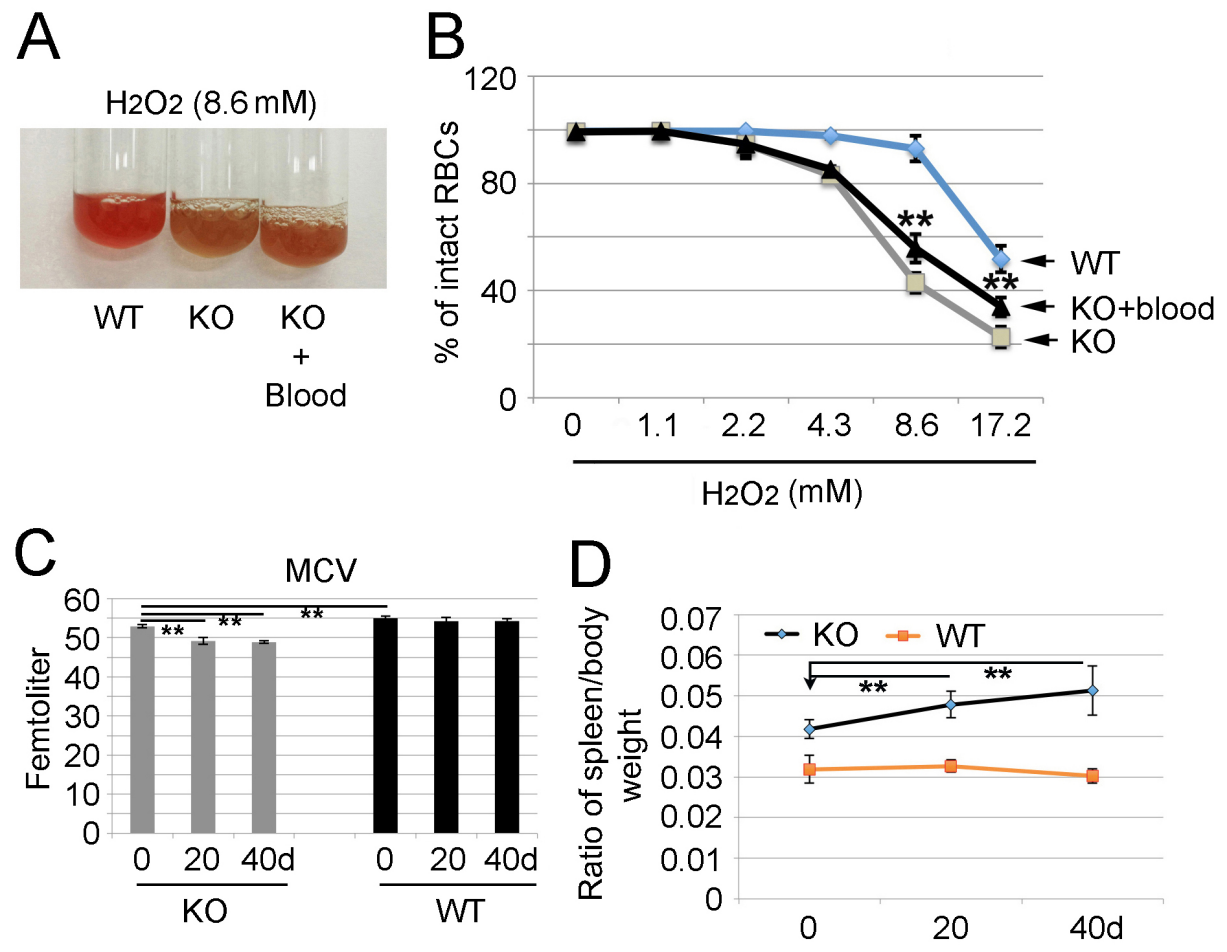
Change of anemic phenotypes of *miR-144/451* KO mice after feeding with wild type blood or special chow diet containing much less miR-451 **(A)** Hydrogen peroxide (H2O2)-induced color change of *miR-144/451* KO blood with and without feeding with WT blood. WT and *miR-144/451* KO blood were used as both negative and positive controls. Note: the color of *miR-144/451* KO blood turned to brown upon exposure to H2O2 (middle tube), however the color of blood from *miR-144/451* KO mice with feeding WT blood was somewhat between red and brown (right tube), suggesting less hemolysis with WT blood feeding. **(B)** Flow cytometric analysis of fractions of intact erythrocytes in *miR-144/451* KO mice after treatment with various concentrations of H2O2. WT blood (light blue line) was used as a control which contained much more intact erythrocytes after treatment with H2O2. Note: blood from *miR-144/451* KO mice fed with WT blood contain significantly increased numbers of intact erythrocytes (black line) compared with the *miR-144/451* KO blood without WT blood feeding (grey line). Five mice from each group were analyzed. ^**^p < 0.01 (*t*-test). **(C)** Measurement of mean corpuscular volume (MCV) in *miR-144/451* KO mice 20 and 40 days after feeding with special chow diet. WT animals were used as control. Note: there is a significant decrease of MCV in *miR-144/451* KO blood cells compared with those in WT animals (at 0 day), and the MCV was even lower in *miR-144/451* KO mice fed with special chow diet. ^**^p < 0.01 (*t*-test). **(D)** Spleen size in *miR-144/451* KO mice fed with special chow diet. The Y-axis shows the ratio of spleen to body weight. The weight of WT spleens was used as normal control. n = 6 for each group. ^**^ p < 0.01 (*t*-test). There is a significant increase of spleen/body weight of *miR-144/451* KO mice fed with special chow diet compared to that of WT mice, indicating a potential compensated erythropoiesis.

As our laboratory and others have previously demonstrated, the protective role of miR-451 in erythropoiesis is due, at least partially, to its ability to mediate the inhibition of the cytoplasmic adaptor protein 14-3-3ζ [20, 27]. *miR-144/451* gene deletion results in elevated 14-3-3ζ level, which sequesters the transcription factor Foxo3 to cytoplasm, thus, the expression of Foxo3 directly-transcribed anti-oxidant genes, catalase (*cat*) and glutathione peroxidases 1 (*gpx1*), are dampened. To investigate whether food-derived miR-451 could protect against erythroid oxidative stress *in vivo*, the anti-oxidant capability of *miR-144/451* KO erythrocytes following WT blood feeding was tested. Upon exposure to H_2_O_2_, a physiological reactive oxygen species (ROS) precursor, the color of *miR-144/451* KO blood turned from red to brown, while the WT control remained red (first two tubes in Figure 6A). However, the color of blood from *miR-144/451* KO mice fed with WT blood was somewhat between red and brown (last tube in Figure 6A). Flow cytometric analysis showed that the color change reflected the rescued hemolysis of *miR-144/451* KO erythrocytes by gavage feeding WT blood (black vs grey lines in Figure 6B). Although the protection was far less than that of the WT control (light blue line in Figure 6B), it still indicates that the elevated miR-451 level in *miR-144/451* KO mice helps to protect erythrocytes from oxidant stress induced hemolysis.

Since mice can uptake miR-451 from a regular chow diet, which contains miR-451 (Figure 5A-5B), we decided to feed mice with a special diet that replaced the fish powder with corn and soybean supplements for 20 and 40 days. As shown in Figure 6C, mean corpuscular volume (MCV) of red blood cells in *miR-144/451* KO mice was significantly lower than that in WT control at day 0 (Figure 6C), which was consistent with our previous finding that *miR-144/451* mutant mice exhibited microcytosis due to the activation of the Cab39/LKB1/AMPK pathway (manuscript submitted). However, the MCV of *miR-144/451* KO mice decreased even more both 20 and 40 days after feeding the mice with a special chow diet (Figure 6C), although there were no significant changes of total red blood cell count (RBC), hemoglobin level (Hb) and hematocrit (HCT) (data not shown), suggesting that a further compensatory hematopoiesis might occur in *miR-144/451* KO mice. To verify this hypothesis, the spleen size was measured and normalized to mouse body weight. There was a significant increase of spleen/body weight ratio of *miR-144/451* KO mice fed with special chow diet compared to that of KO mice fed with regular mouse chow diet (Figure 6D), indicating a compensation in hematopoiesis in *miR-144/451* KO mice after feeding the less dietary miR-451.

This data suggest that miR-451, even at a very low level, can significantly reduce hemolysis of *miR-144/451* KO erythroid cells. Without the diet-derived miR-451, mutant red blood cells are more susceptible to oxidant stress-induced injury. To investigate further whether trace amount of miR-451 is sufficient to inhibit gene expression, the 3′UTR of *Ywhaz/14-3-3ζ* mRNA was linked to the protein-coding region of luciferase cDNA, and the luciferase reporter was transfected along with different amounts of *miR-451* expression construct into S17, a stromal cell line derived from mouse bone marrow [28]. The expression levels of *miR-451* 48 hours after transfection were confirmed using qRT-PCR (Supplementary Figure 4A). Fusion of the *Ywhaz/14-3-3ζ* 3′UTR to luciferase cDNA, along with as low as 0.025 ng/ml of miR-451 retroviral vector, dramatically decreased enzymatic activity in transfected S17 cells, presumably reflecting decreased luciferase protein expression (Supplementary Figure 4B). miR-451 at the concentration of 0.25 ng/ml reached the maximum inhibition of luciferase activity (Supplementary Figure 4B). Thus, miR-451 even at a very low concentration inhibits 14-3-3ζ protein expression by interacting directly with its 3′UTR of mRNA.

### Exogenous miR-451 existing in *miR-144/451* KO mice enhances anti-oxidant activity *in vivo* via increasing the activity of Foxo3 pathway

**Figure 7:**
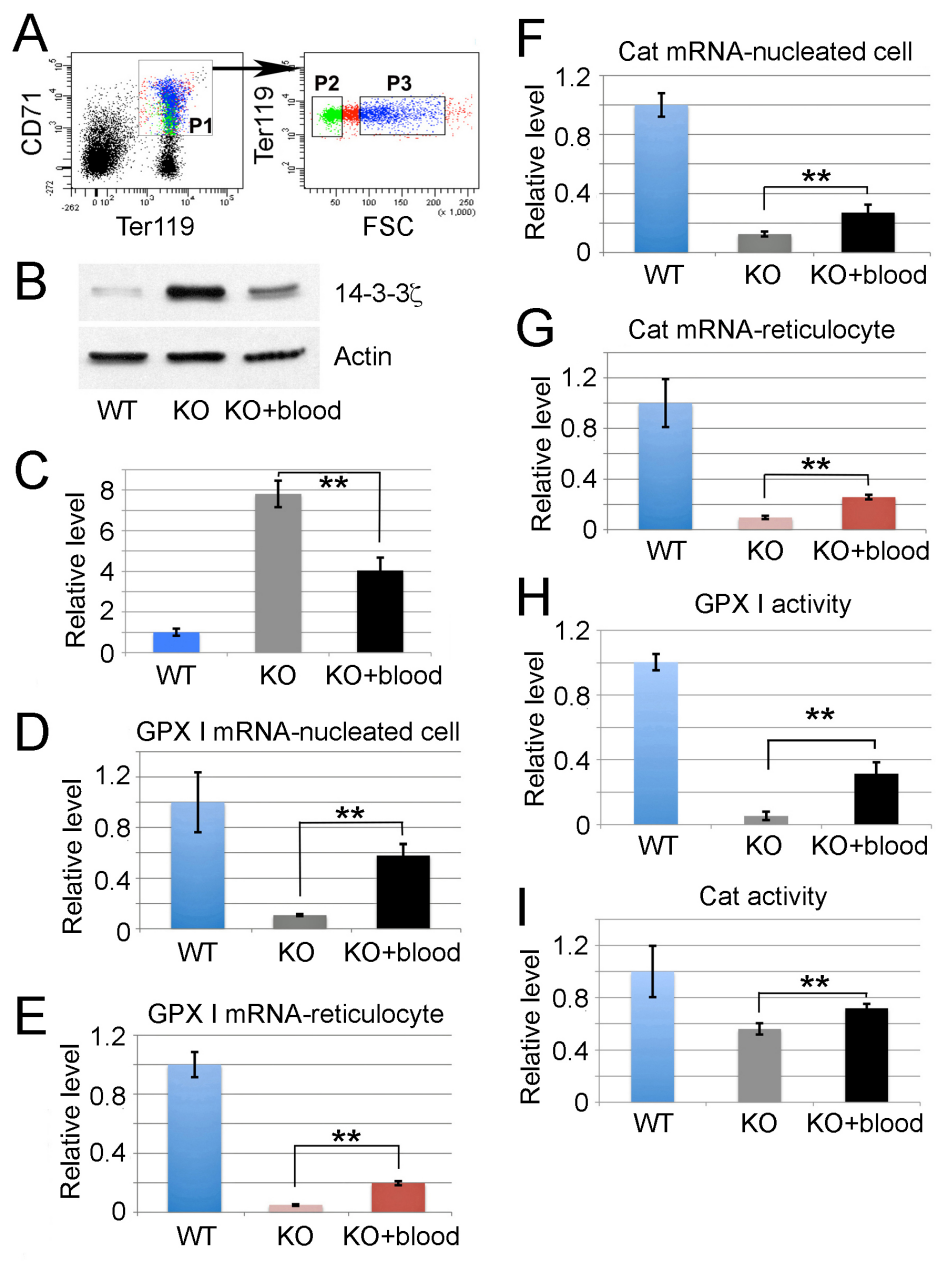
Exogenous miR-451 existing in *miR-144/451* KO mice enhances anti-oxidant activity *in vivo* via increasing the activity of Foxo3 pathway **(A)** Bone marrow erythroblasts were fractionated according to the developmental markers Ter119, CD71, and forward scatter (FSC) intensity. Ter119^+^/CD71^+^/FSC^hi^ cells represent nucleated erythroblasts while Ter119^+^/CD71^+^/FSC^low^ cells represent reticulocytes. **(B)** Western blot showing repressed 14-3-3ζ protein expression in *miR-144/451* KO bone marrow erythroblasts after feeding with WT blood relative to control erythroblasts from *miR-144/451* KO bone marrow without feeding WT blood. Lysate from WT bone marrow erythroblasts was used as negative control. **(C)** Quantitative analysis of western blot signals of 14-3-3ζ protein from 3 independent experiments. ^**^p < 0.01 (*t*-test). **(D-E)** qRT–PCR analysis to quantify *gpx1* expression at transcription level in nucleated erythroblasts (D) and reticulocytes (E) from *miR-144/451* KO mice with and without feeding WT blood. WT nucleated erythroblasts and reticulocytes were used as positive controls. Representative studies from three mice of each group are shown. ^**^p < 0.01 (*t*-test). **(F-G)** The transcription level of *cat* gene in nucleated erythroid cells (F) and reticulocytes (G) are shown in the same experimental setting. ^**^p < 0.01 (*t*-test). **(H-I)** Increased glutathione peroxidase 1 (GPX I) (H) and catalase (I) activities in *miR-144/451* KO erythrocytes fed with WT blood compared with the erythrocytes from untreated *miR-144/451* KO controls. Relative GPX I and catalase activities from equal numbers of erythrocytes are shown. n = 6. ^**^ P < 0.01 (*t*-test).

To investigate the effects of exogenous miR-451 on the 14-3-3ζ/Foxo3 pathway activity, *miR-144/451* KO mice were gavage fed with fresh WT blood twice a day for three weeks. The mouse bone marrow erythroblasts were then purified according to expression of developmental markers CD71, Ter119, and FSC (Figure 7A). The protein level of 14-3-3ζ, previously confirmed as a direct target of miR-451 by our laboratory [20], and two downstream anti-oxidant enzymes (Cat and Gpx1) were examined. As shown in Figure 7B and 7C, the 14-3-3ζ protein level in mouse bone marrow erythroid cells following WT blood feeding was dramatically reduced. The mRNA levels of *gpx1* and *cat*, both of which are directly transcribed by Foxo3 whose activity was negatively regulated by 14-3-3ζ, were elevated in both nucleated erythroid blasts and reticulocytes although the levels were still much lower than that of WT control cells (Figure 7D-7G). Consistently, the activities of *gpx1* and *cat* encoded enzymes were also enhanced by WT blood feeding (Figure 7H-7I). This data clearly indicates that the extra level of miR-451 derived from food intake regulates the 14-3-3ζ/Foxo3 signaling pathway.

## DISCUSSION

A recent study has shown that rice-derived miR-168a can accumulate in the blood and regulate the gene expression of humans or animals [2]. This suggests that miRNAs orally ingested from food, drink, nutrient supplements or drugs might be used as a novel source of reagents, which could be beneficial for human and animal health. At the same time, this report raises the safety concern that many food products, including bio-engineered food products, from plants or animals could pose health risks to human. Due to its significant relevance to our daily life, this profound discovery immediately attracted many scientists to further investigate the key question: is it true that miRNAs are capable of gene regulation through daily ingestion? As miRagen Therapeutics and Monsanto clarify—the initial claims of oral delivery and gene regulatory effect of food miR-168a in mammals may prove to be overstated [16], a verdict supported by several negative experimental reports. Despite the negative findings there are several other studies showing that gene regulation by miRNA from food is possible. For example, Zhou et al. detected plant miRNAs in the sera and tissue of honeysuckle-fed mice [29]. Mlotshwa et al. engineered plants to express artificial miRNAs that can silence essential human genes to treat cancer [30]. Cavalieri et al. found that food-derived plant miRNA function in recipient cells in a sequence-independent manner by binding to TLR3 of dendritic cells [31].

miR-144/451’s expression in white blood cells or any other non-erythroid cell is negligible. On the other hand they are the most abundantly expressed miRNAs in erythroid cells, and therefore blood contains very high levels of miR-144/151. This makes our *miR-144/451* KO model particularly special. In this study we utilize this mouse model to show that oral ingestion of WT blood leads to increased miR-451 and miR-144 levels in the circulating blood of *miR-144/451* KO mice. Furthermore, the increased mature miR-451 molecules in *miR-144/451* KO blood enhance the anti-oxidant ability of erythroid cells by suppressing 14-3-3ζ and thus allowing the transcription of two anti-oxidant genes—*cat* and *gpx1*. Our data clearly indicates that miR-451 in food can pass through the digestive system to enter the bloodstream and protect the red blood cells.

Another interesting finding in our study is that although *miR-144/45*1 genes have been knocked out, blood in *miR-144/451* KO mice fed with regular chow diet still contains low levels of miR-451. This has been confirmed by sequencing the PCR products (see Figure 1E). This miRNA mainly comes from the regular mouse chow diet that contains animal-derived, presumably fish-derived miR-451. Reducing miR-451 level in *miR-144/451* KO blood, by feeding a special food that contains much less miR-451, accelerates microcytosis. This suggests that if a miRNA is highly conserved, the miRNA gene KO animals might still have low levels of exogenous miRNAs that inhibit their target genes and affect animal functions. This finding should concern the scientists who use miRNA KO models to study gene functions. It is particularly true for miRNAs including miR-451 whose levels could be very low in KO animals but could still have a profound inhibitory effect on their target genes. In this study we show that miR-144/451 producing retroviruses at very low concentrations can sufficiently suppress the expression of target genes, highlighting that high levels of miRNAs are not necessary for phenotype change of the target cells or tissues.

In mammals, miRNAs have been found in serum, plasma, urine, saliva, and even breast milk [17, 32-37], suggesting that miRNAs are stable in those body fluids. It has been reported that some miRNAs are resistant to acidic conditions, high temperature and RNase cleavage [38, 39]. Consistent to these findings, our experiments have confirmed that miR-451 is extremely stable under highly acidic, alkaline and hot conditions. However, miR-451 can be easily degraded by RNase though the stability is higher than that of U6 control RNA. This indicates that RNase, which usually exists in saliva or digested food in gastric fluids, can biologically block miRNAs into the bloodstream. This explains, at least in part, that oral administration of WT blood allows only limited access of miR-451 and miR-144 to the circulation of *miR-144/451* KO animals. Packing in microvesicles or similar systems might be a way to protect miRNAs from degradation by RNase [40].

Despite more emerging evidence that food-derived miRNAs can pass through the GI tract to circulating blood, the underlining mechanisms are still not clear. Recently, several lines of evidence suggest that to be able to enter the circulation to affect the functions of recipient animals, food-derived miRNAs must overcome numerous challenges [41]. These challenges include the harsh conditions in the stomach and intestine, being blocked by the lumen of intestinal epithelium [42, 43], insufficient protection by microvesicles [40, 44, 45] and the components of RNA-induced silencing complex [46] and the high-density lipoprotein [47]. Lastly, on reaching the final recipient cells in organs, miRNAs must then enter the cells themselves. This scenario highlights the proposed difficulty for miRNAs to fully access the target cells through the GI tract to perform their functions *in vivo*. Recently, Snow et al. evidenced that no robust levels of miR-21 are detected in miR-21 null mice after consuming miR-21 [16]. In current study, we found that the mouse digestive system allows different degrees of miRNA uptake (miR-451 vs. miR-15a). These findings suggest that miRNAs in food may undergo selective absorption and the properties of dietary miRNAs (e.g., sequence, nucleotide composition, modification, packaging and protein-association) all contribute to the efficacy of uptake, yet the exact mechanisms are still unclear.

In conclusion, we use a very simple and an easily reproduced animal model, the *miR-144/451* KO model, to show that miR-451 is sufficiently detected in the circulating blood of *miR-144/451* KO mice fed with regular chow diet that contains well conserved miR-451. Whereas feeding *miR-144/451* KO mice with a chow diet containing less miR-451 reduces the miR-451 level in *miR-144/451* KO blood. In addition, gavage-feeding *miR-144/451* mutant mice with WT blood cells that express abundant miR-144/451 elevates both miR-451 and miR-144 levels in *miR-144/451* KO blood. Moreover, alteration of erythroid phenotypes can be accomplished by either lowering (special chow diet) or by increasing (gavage-feeding WT blood) the miR-451 level in *miR-144/451* KO blood. We also use a luciferase report assay to show that a limited amount of miR-451 is enough to inhibit gene expression. These results clearly indicate that ingestion of at least some miRNAs, such as miR-451, affects the function of the consumers, suggesting that regulatory information, whether detrimental to or beneficial for health, could be inherited from ingested reagents including everyday food products, nutrient supplements and small RNA interfering drugs. Our finding is also a valid endeavor considering the significant impact of trace amount of absorbed miRNAs from chow diet on the gene regulation in miRNA study using miRNA KO animals.

## MATERIALS AND METHODS

### Animal feeding studies

All animal experiments were approved by the Animal Care and Use Committee of the Yangzhou University School of Medicine. *miR-144/451* KO mice lacking of a 388 bp segment of genomic DNA containing the bicistronic *miR-144* and *miR-451* locus was described in our published work [20]. *lnk* gene KO mice were kindly provided by Tony Pawson [24] (Samuel Lunenfeld Research Institute, Toronto, Ontario, Canada) and maintained in our laboratory since 2012. *miR-15a/16-1* KO mice were kindly provided by Dr. Xiaoqin Jia (Yangzhou University School of Medicine) and were originally from Jackson Laboratory. Regular mouse chow diet and customized rodent diets devoid of fish powder were purchased from Trophic Animal Feed High-Tec Co., Ltd, China. Blood from WT mice was collected to EDTA-coated blood collection tubes via retro-orbital bleeding. 200 μl blood per mouse was delivered directly into the *miR-144/451* KO mouse stomach via oral gavage feeding using a bulb tipped gastric gavage needle. Blood was collected from these recipient mice at different time points for RNA extraction. For *lnk* and *miR-15a/16-1* experiments, spleens from WT mice were cut into small pieces and dispersed for making cell suspensions. After red blood cells were lysed, splenocytes were suspended in 20x10^6^/200 μl phosphate buffer saline (PBS). 200 μl such splenocyte suspension per mouse was gavage-fed to the *lnk* or *miR-15a/16-1* KO mice. All gavage feeding studies were repeated at least three times and the results shown in this report are representative of 5-6 mice in each group.

### Exosome isolation

200 μl WT blood was gavage-fed to *miR-144/451* KO mice and 6 hours later 100 μl blood per mouse was collected via retro-orbital bleeding. Blood was centrifuged at 3000 rpm for 5 min to remove cells and large debris. Exosomes were then isolated from the plasma using QIAGEN’s exoRNeasy Serum/Plasma Starter Kit (Catalog ID 77023, QIAGEN).

### Quantitative real time PCR (qRT-PCR) analysis

Total RNA was extracted from each 200 μl blood sample using Trizol Plus reagent from Invitrogen, dissolved in 50 μl of water, treated with deoxyribonuclease (DNase), and then 2.5 μl total RNA was converted to cDNAs in 20 μl total reaction volume using different kits according to the manufacturer’s instructions. For isolation of total RNAs from chow diets, the mouse diets were finely grounded and the chow powders were used to extract RNA using Trizol reagent. To compare the miRNA level in cooked blood with that in fresh blood, blood from mouse, chicken and pig was boiled in 95°C water bath for 10 min and the cooked blood was grounded for RNA extraction. All-in-One™ miRNA qRT-PCR Detection kit (GeneCopoeia, Rockville, MD) and TaqMan microRNA Assay Kit obtained from Life Technologies (Grand Island, NY) were used to detect miRNA expression. 2 μl of the 100 μl reverse transcription (RT) product (1:5 diluted) was used for each quantitative PCR reaction. In all qRT-PCR experiments, small nuclear RNA U6 was used as an internal loading control. For *lnk* mRNA detection, the PrimeScript™ First Strand cDNA synthesis kit (Takara Bio. Inc, Japan) was used to make cDNA. Mouse beta-actin was used as sample loading control. Synthetic miR-451 oligonucleotides were purchased from Takara Bio. Inc. and diluted in water as stocking solution at the concentration of 32 pg/ml, with estimated 2.74112x10^9^ miR-451 copies/ml. 2.5 μl of such stocking solution and a serial of 1:2 diluted solutions were used for reverse transcription reactions in 20 μl total volume. Water without template was used as negative control. The 20 μl PCR reaction using 2 μl RT product as template was performed to detect cDNA levels using an ABI 7900 Sequence Detection System with the SYBR Green PCR reagent kit (Applied Biosystems, Foster City, CA). Sequences of the primers used for qRT-PCR are provided in Supplementary Table 1. All the reactions were conducted in triplicate. Standard delta-delta-Ct method was used to calculate relative levels of RNAs. The copy number equivalent to 1 pg of miR-451 was calculated according to DNA/RNA Copy Number Calculator (http://www.endmemo.com/bio/dnacopynum.php). The copy number per μl of blood was calculated based on the input total RNA and the standard curve of synthetic miR-451. For example, copy number of miR-451 per μl of *miR-144/451* KO blood at 30 C_T_ = [21415 copies x (100 μl total RT products / 2 μl RT products used for PCR) x (50 μl total RNA from 200 μl blood / 2.5 μl RNA used for RT)]/200 μl blood = 107035 copies/μl.

### Dual-luciferase reporter assay

To make miR-451 expression vector, genomic fragments encompassing mouse *miR-451* (272 bp) were cloned into MSCV-PIG retroviral vector by PCR using the primers engineered with XhoI and EcoRI restriction sites (sequences listed in Supplementary Table 1). The 3’ UTR fragment of Ywhaz/14-3-3ζ mRNA was amplified by PCR and cloned into the EcoRI and XbaI sites of the modified pGL3 luciferase reporters pGL3-BS (Promega Corporation, Madison, WI, USA) as shown previously [20]. Co-transfection of Renilla vectors and Ywhaz/14-3-3ζ 3’-UTR luciferase reporter plasmid to S17 mouse bone marrow stromal cells with or without the miR-451 retroviral vector was performed using Lipofectamine 2000 (Invitrogen Life Technologies). After 24 hours, dual-luciferase activities were measured using the Dual-Luciferase® reporter assay kit (Promega Corporation) and a Veritas Microplate Luminometer (Promega) according to the manufacturer’s instructions.

### Fluorescent activated cell sorting (FACS)

Expression of red cell surface markers was analyzed on a FACS Calibur instrument (Becton Dickinson, Lincoln Park, NJ, USA). Cells were washed and re-suspended in PBS containing 0.1% BSA (FACS buffer), and stained with fluorescence-labeled antibodies. Data analyses were performed with FlowJ software (TreeStar). Flow cytometry antibodies were purchased from BD Biosciences: APC rat anti-mouse Ter119 (cat^#^ 557909) and PE or FITC labeled rat anti-mouse CD71 (cat^#^ 553267 or 553266, respectively, San Jose, CA, USA). Erythroid subpopulations (nucleated erythroblasts and reticulocytes) were sorted using a FACSAria cytometer (BD Biosciences) based on CD71/Ter119 expressions. CD71^+^/Ter119^+^/FSC^high^ cells were defined as nucleated cells, whereas CD71^+^/Ter119^+^/FSC^low^ cells were considered reticulocytes [20]. To measure hydrogen peroxide (H2O2)-induced red cell lyses, whole blood diluted at 1:500 in PBS was incubated with different concentrations of H2O2 at room temperature for 5 minutes, shook vigorously for 15 seconds, and analyzed immediately on forward scatter channel (FSC) and side scatter channel (SSC) using flow cytometry.

### Hematological studies

Blood from adult mice was sampled retro-orbitally and anti-coagulated with EDTA. Complete blood counts were determined using the Hemavet HV950FS analyzer (Drew Scientific, Dallas, TX, USA). Reticulocyte counts were done using Ter119/CD71 antibody staining and analyzed on a FACS Calibur (Becton Dickinson).

### Western blot analysis

Protein lysates from purified erythroblasts were prepared using cell lysis buffer with 1:500 protease inhibitor mixture (Sigma-Aldrich). Protein concentrations were determined using a Pierce bicinchoninic acid assay kit (Thermo Scientific, Rockford, IL, USA), and 10 μg of protein were resolved by 10% SDS-PAGE gel. After being transferred to polyvinylidene fluoride membranes (Whatman), proteins were detected using primary antibodies and HRP-conjugated secondary antibodies. The following antibodies for western blots were used: β-actin (clone AC-15, A3854, Sigma-Aldrich) and 14-3-3ζ (cat^#^ sc-1019, Santa Cruz Biotechnology). Horseradish peroxidase-conjugated anti-β-actin antibody was used as a loading control. Western blots were developed using SuperSignal West Pico chemiluminescent reagent. Western blotting secondary antibodies, markers, and reagents were obtained from Thermo Scientific.

### Measurement of catalase and glutathione peroxidase 1 activity

Erythrocyte catalase activity was determined using the Catalase Assay Kit (Calbiochem) and normalized to hemoglobin concentration (OD570 nm) within cell lysates. Glutathione Peroxidase Cellular Activity Assay Kit (Sigma-Aldrich) was used for measurement of glutathione peroxidase activity in mature red cell lysates according to manufacturer’s instruction.

### RNA treatment

To mimic the acidic environment of the stomach, red blood cells from WT mice were incubated at 37°C for two hours with either an acidic solution whose pH was adjusted to 2.5 or a simulated gastric fluid (SGF) [26]. To mimic the alkaline environment of mouse blood, RNA from WT mouse blood was adjusted with a NaOH solution to pH 8.5 or was added to a simulated intestine fluid (SIF) [26] for two hours at 37°C. For RNase treatment, RNA samples were adjusted to 200 μl with different concentrations of ribonuclease A (RNase A, Sigma) and incubated at room temperature for 30 minutes. RNase A was heat inactivated at 70°C for 15 minutes.

## SUPPLEMENTARY MATERIALS FIGURES AND TABLE



## References

[1] Petrick JS, Brower-Toland B, Jackson AL, Kier LD (2013). Safety assessment of food and feed from biotechnology-derived crops employing RNA-mediated gene regulation to achieve desired traits: a scientific review. Regul Toxicol Pharmacol.

[2] Zhang L, Hou D, Chen X, Li D, Zhu L, Zhang Y, Li J, Bian Z, Liang X, Cai X, Yin Y, Wang C, Zhang T (2012). Exogenous plant MIR168a specifically targets mammalian LDLRAP1: evidence of cross-kingdom regulation by microRNA. Cell Res.

[3] Liang G, Zhu Y, Sun B, Shao Y, Jing A, Wang J, Xiao Z (2014). Assessing the survival of exogenous plant microRNA in mice. Food Sci Nutr.

[4] Lukasik A, Zielenkiewicz P (2014). In silico identification of plant miRNAs in mammalian breast milk exosomes—a small step forward?. PLoS One.

[5] Yang J, Farmer LM, Agyekum AA, Hirschi KD (2015). Detection of dietary plant-based small RNAs in animals. Cell Res.

[6] Shu J, Chiang K, Zempleni J, Cui J (2015). Computational characterization of exogenous microRNAs that can be transferred into human circulation. PLoS One.

[7] Philip A, Ferro VA, Tate RJ (2015). Determination of the potential bioavailability of plant microRNAs using a simulated human digestion process. Mol Nutr Food Res.

[8] Chin AR, Fong MY, Somlo G, Wu J, Swiderski P, Wu X, Wang SE (2016). Cross-kingdom inhibition of breast cancer growth by plant miR159. Cell Res.

[9] Yang J, Hotz T, Broadnax L, Yarmarkovich M, Elbaz-Younes I, Hirschi KD (2016). Anomalous uptake and circulatory characteristics of the plant-based small RNA MIR2911. Sci Rep.

[10] Chen X, Dai GH, Ren ZM, Tong YL, Yang F, Zhu YQ (2016). Identification of dietetically absorbed rapeseed (Brassica campestris L.) bee pollen microRNAs in serum of mice. Biomed Res Int.

[11] Baier SR, Nguyen C, Xie F, Wood JR, Zempleni J (2014). MicroRNAs are absorbed in biologically meaningful amounts from nutritionally relevant doses of cow milk and affect gene expression in peripheral blood mononuclear cells, HEK-293 kidney cell cultures, and mouse livers. J Nutr.

[12] Liang H, Zhang S, Fu Z, Wang Y, Wang N, Liu Y, Zhao C, Wu J, Hu Y, Zhang J, Chen X, Zen K, Zhang CY (2015). Effective detection and quantification of dietetically absorbed plant microRNAs in human plasma. J Nutr Biochem.

[13] Zhang Y, Wiggins BE, Lawrence C, Petrick J, Ivashuta S, Heck G (2012). Analysis of plant-derived miRNAs in animal small RNA datasets. BMC Genomics.

[14] Dickinson B, Zhang Y, Petrick JS, Heck G, Ivashuta S, Marshall WS (2013). Lack of detectable oral bioavailability of plant microRNAs after feeding in mice. Nat Biotechnol.

[15] Witwer KW, McAlexander MA, Queen SE, Adams RJ (2013). Real-time quantitative PCR and droplet digital PCR for plant miRNAs in mammalian blood provide little evidence for general uptake of dietary miRNAs: limited evidence for general uptake of dietary plant xenomiRs. RNA Biol.

[16] Snow JW, Hale AE, Isaacs SK, Baggish AL, Chan SY (2013). Ineffective delivery of diet-derived microRNAs to recipient animal organisms. RNA Biol.

[17] Title AC, Denzler R, Stoffel M (2015). Uptake and Function Studies of Maternal Milk-derived MicroRNAs. J Biol Chem.

[18] Micó V, Martín R, Lasunción MA, Ordovás JM, Daimiel L (2016). Unsuccessful detection of plant microRNAs in beer, extra virgin olive oil and human plasma after an acute ingestion of extra virgin olive oil. Plant Foods Hum Nutr.

[19] Tosar JP, Rovira C, Naya H, Cayota A (2014). Mining of public sequencing databases supports a non-dietary origin for putative foreign miRNAs: underestimated effects of contamination in NGS. RNA.

[20] Yu D, dos Santos CO, Zhao G, Jiang J, Amigo JD, Khandros E, Dore LC, Yao Y, D’Souza J, Zhang Z, Ghaffari S, Choi J, Friend S (2010). miR-451 protects against erythroid oxidant stress by repressing 14-3-3zeta. Genes Dev.

[21] Dore LC, Amigo JD, Dos Santos CO, Zhang Z, Gai X, Tobias JW, Yu D, Klein AM, Dorman C, Wu W, Hardison RC, Paw BH, Weiss MJ (2008). A GATA-1-regulated microRNA locus essential for erythropoiesis. Proc Natl Acad Sci USA.

[22] Zhang L, Flygare J, Wong P, Lim B, Lodish HF (2011). miR-191 regulates mouse erythroblast enucleation by down-regulating Riok3 and Mxi1. Genes Dev.

[23] Pekarsky Y, Croce CM (2015). Role of miR-15/16 in CLL. Cell Death Differ.

[24] Velazquez L, Cheng AM, Fleming HE, Furlonger C, Vesely S, Bernstein A, Paige CJ, Pawson T (2002). Cytokine signaling and hematopoietic homeostasis are disrupted in Lnk-deficient mice. J Exp Med.

[25] McConnell EL, Basit AW, Murdan S (2008). Measurements of rat and mouse gastrointestinal pH, fluid and lymphoid tissue, and implications for in-vivo experiments. J Pharm Pharmacol.

[26] Fu TJ, Abbott UR, Hatzos C (2002). Digestibility of food allergens and nonallergenic proteins in simulated gastric fluid and simulated intestinal fluid-a comparative study. J Agric Food Chem.

[27] Patrick DM, Zhang CC, Tao Y, Yao H, Qi X, Schwartz RJ, Jun-Shen Huang L, Olson EN (2010). Defective erythroid differentiation in miR-451 mutant mice mediated by 14-3-3zeta. Genes Dev.

[28] Collins LS, Dorshkind K (1987). A stromal cell line from myeloid long-term bone marrow cultures can support myelopoiesis and B lymphopoiesis. J Immunol.

[29] Zhou Z, Li X, Liu J, Dong L, Chen Q, Liu J, Kong H, Zhang Q, Qi X, Hou D, Zhang L, Zhang G, Liu Y (2015). Honeysuckle-encoded atypical microRNA2911 directly targets influenza A viruses. Cell Res.

[30] Mlotshwa S, Pruss GJ, MacArthur JL, Endres MW, Davis C, Hofseth LJ, Peña MM, Vance V (2015). A novel chemopreventive strategy based on therapeutic microRNAs produced in plants. Cell Res.

[31] Cavalieri D, Rizzetto L, Tocci N, Rivero D, Asquini E, Si-Ammour A, Bonechi E, Ballerini C, Viola R (2016). Plant microRNAs as novel immunomodulatory agents. Sci Rep.

[32] Lawrie CH, Gal S, Dunlop HM, Pushkaran B, Liggins AP, Pulford K, Banham AH, Pezzella F, Boultwood J, Wainscoat JS, Hatton CS, Harris AL (2008). Detection of elevated levels of tumour-associated microRNAs in serum of patients with diffuse large B-cell lymphoma. Br J Haematol.

[33] Park NJ, Zhou H, Elashoff D, Henson BS, Kastratovic DA, Abemayor E, Wong DT (2009). Salivary microRNA: discovery, characterization, and clinical utility for oral cancer detection. Clin Cancer Res.

[34] Hanke M, Hoefig K, Merz H, Feller AC, Kausch I, Jocham D, Warnecke JM, Sczakiel G (2010). A robust methodology to study urine microRNA as tumor marker: microRNA-126 and microRNA-182 are related to urinary bladder cancer. Urol Oncol.

[35] Kosaka N, Izumi H, Sekine K, Ochiya T (2010). microRNA as a new immune-regulatory agent in breast milk. Silence.

[36] Chen X, Gao C, Li H, Huang L, Sun Q, Dong Y, Tian C, Gao S, Dong H, Guan D, Hu X, Zhao S, Li L (2010). Identification and characterization of microRNAs in raw milk during different periods of lactation, commercial fluid, and powdered milk products. Cell Res.

[37] Alsaweed M, Hartmann PE, Geddes DT, Kakulas F (2015). MicroRNAs in breastmilk and the lactating breast: potential immunoprotectors and developmental regulators for the infant and the mother. Int J Environ Res Public Health.

[38] Mitchell PS, Parkin RK, Kroh EM, Fritz BR, Wyman SK, Pogosova-Agadjanyan EL, Peterson A, Noteboom J, O’Briant KC, Allen A, Lin DW, Urban N, Drescher CW (2008). Circulating microRNAs as stable blood-based markers for cancer detection. Proc Natl Acad Sci USA.

[39] Chen X, Ba Y, Ma L, Cai X, Yin Y, Wang K, Guo J, Zhang Y, Chen J, Guo X, Li Q, Li X, Wang W (2008). Characterization of microRNAs in serum: a novel class of biomarkers for diagnosis of cancer and other diseases. Cell Res.

[40] Zhang Y, Liu D, Chen X, Li J, Li L, Bian Z, Sun F, Lu J, Yin Y, Cai X, Sun Q, Wang K, Ba Y (2010). Secreted monocytic miR-150 enhances targeted endothelial cell migration. Mol Cell.

[41] Jiang M, Sang X, Hong Z (2012). Beyond nutrients: food-derived microRNAs provide cross-kingdom regulation. BioEssays.

[42] Feinberg EH, Hunter CP (2003). Transport of dsRNA into cells by the transmembrane protein SID-1. Science.

[43] Duxbury MS, Ashley SW, Whang EE (2005). RNA interference: a mammalian SID-1 homologue enhances siRNA uptake and gene silencing efficacy in human cells. Biochem Biophys Res Commun.

[44] Kosaka N, Iguchi H, Yoshioka Y, Takeshita F, Matsuki Y, Ochiya T (2010). Secretory mechanisms and intercellular transfer of microRNAs in living cells. J Biol Chem.

[45] Pegtel DM, Cosmopoulos K, Thorley-Lawson DA, van Eijndhoven MA, Hopmans ES, Lindenberg JL, de Gruijl TD, Würdinger T, Middeldorp JM (2010). Functional delivery of viral miRNAs via exosomes. Proc Natl Acad Sci USA.

[46] Turchinovich A, Weiz L, Langheinz A, Burwinkel B (2011). Characterization of extracellular circulating microRNA. Nucleic Acids Res.

[47] Vickers KC, Palmisano BT, Shoucri BM, Shamburek RD, Remaley AT (2011). MicroRNAs are transported in plasma and delivered to recipient cells by high-density lipoproteins. Nat Cell Biol.

