# Supplementary material for: Dietary miR-451 protects erythroid cells from oxidative stress via increasing the activity of Foxo3 pathway
Source: Oncotarget. 2017 Nov 10;8(63):107109–24. doi: 10.18632/oncotarget.22346 (PMC5739800; doi:10.18632/oncotarget.22346)
Supplement: Supplementary file 1 [file oncotarget-08-107109-s001.pdf]

Dietary miR-451 protects erythroid cells from oxidative stress via increasing the activity of Foxo3 pathway

SUPPLEMENTARY MATERIALS

Supplementary Table 1: Sequences of primers

Name		Sequence	Length of PCR product (bp)
miR-451	Forward	5'-AAACCGTTACCATTACTGAGTT-3'	
miR-144	Forward	5'-TACAGTATAGATGATGTACT-3'	
miR-15a	Forward	5'-TAGCAGCACATAATGGTTTGTG-3'	
u6	Forward	5'-CGCTTCGGCAGCACATATAC-3'	
cat	Forward	5'-TCTGGGACTTCTGGAGTCTT-3'	114
	Reverse	5'-AGGTGTGTGATCCATAGCCA-3'	
gpx1	Forward	5'-TGCAATCAGTTCGGACACCA-3'	136
	Reverse	5'-GAGCCTTCTCACCATTCACT-3'	
lnk	Forward 1	5'-GACAACCTCTACACCTTTG-3'	51
	Reverse 1	5'-AGATGATGTCTGTCTGGTC-3'	
lnk	Forward 2	5'-GACAACCTCTACACCTTTG-3'	95
	Reverse 2	5'-TCTGCCAGCCATGAGTTCA-3'	
lnk	Forward 3	5'-GACAACCTCTACACCTTTG-3'	152
	Reverse 3	5'-GAAAGAGGTAACTCGGTGT-3'	
miR-451 gene cloning	Forward	5'-ACTGCTCGAGCCCCTGGGTAC CCCACCTCCAGAG-3' (Xho I)	272
	Reverse	5'-CAGTGAATTCTTAATTAAAAAGAAAATGTAC CCTTTCCCCCAACC-3' (EcoR I)	

Universal Adaptor PCR Primer (GeneCopoeia, Rockville, MD, USA) was used as the reverse primer for the detection of all miRNAs and U6.

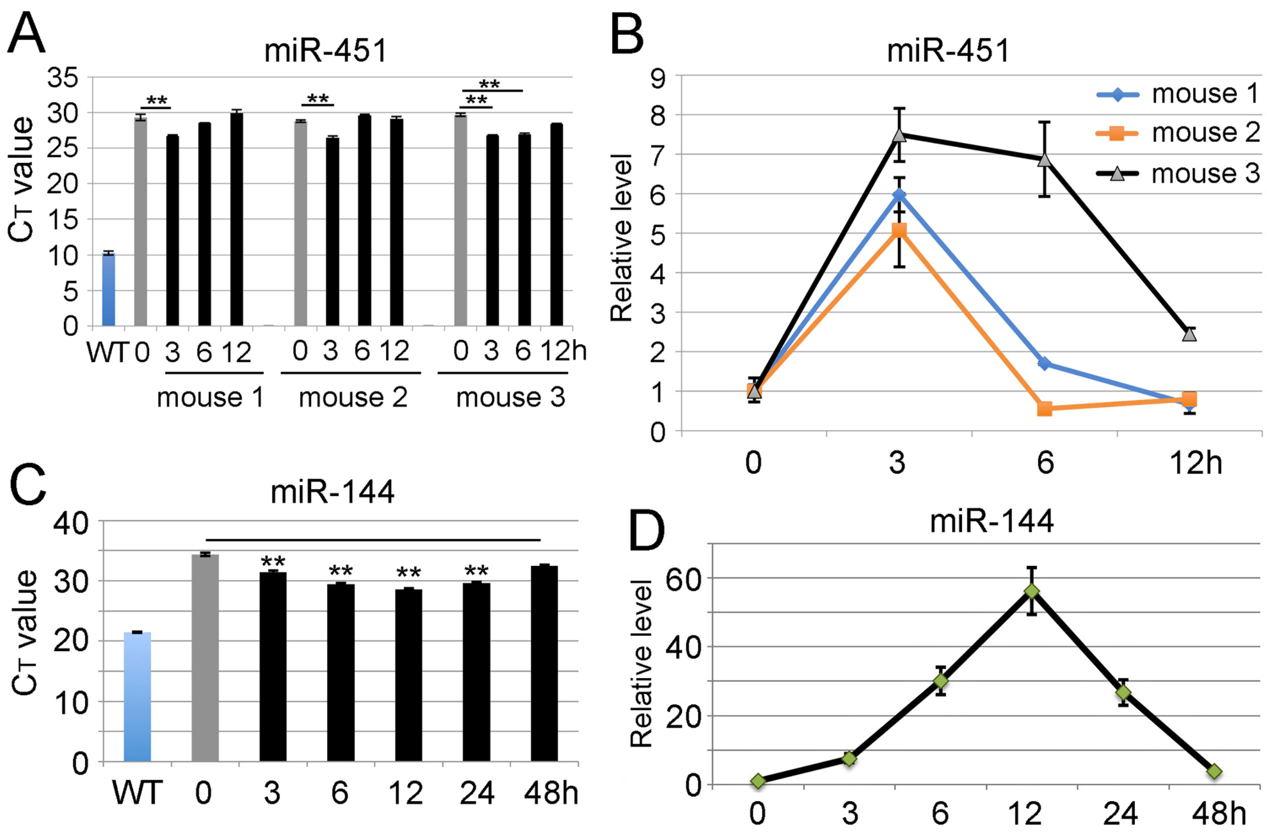

Supplementary Figure 1: Oral uptake of miR-451 and miR-144 increases the levels of miRNA in peripheral blood of *miR-144/451* KO mice. (A) C_T value from quantitative PCR analysis of miR-451 levels in peripheral blood of *miR-144/451* KO mice after ingestion of synthetic miR-451. **(B)** Relative levels of miR-451. X-axis shows hours after feeding synthetic miR-451. miR-451 level in WT mice was used as positive control. Data is from three independent experiments. **(C)** C_T value from qRT-PCR analysis of miR-144 levels in peripheral blood of *miR-144/451* KO mice after ingestion of wild type blood. **(D)** Relative levels of miR-144. miR-144 level in WT mice was used as positive control. $^{**}p < 0.01$ (*t*-test).

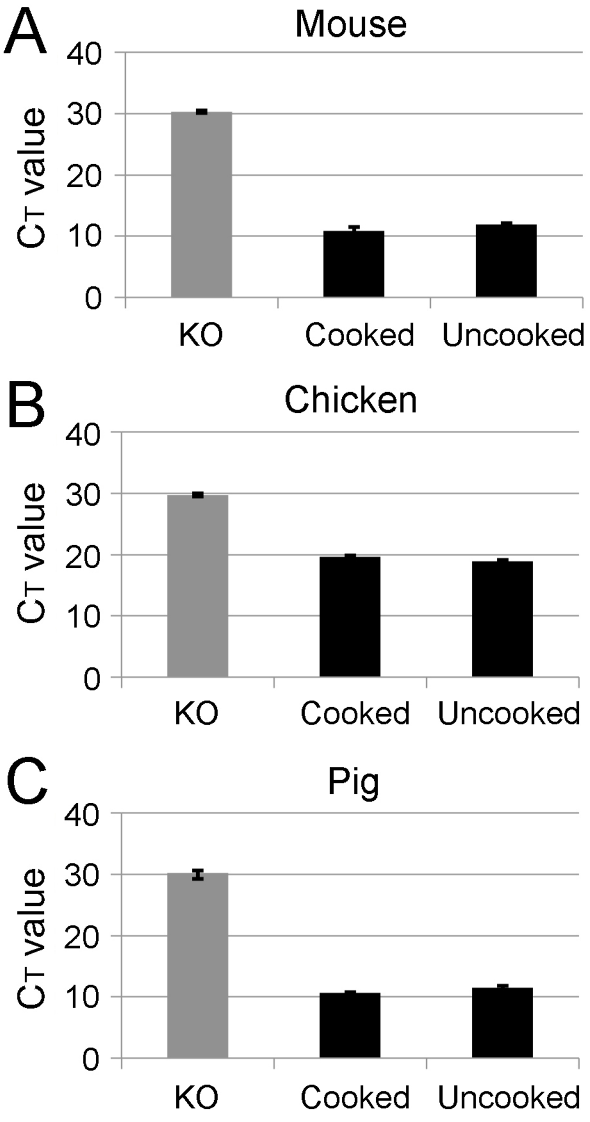

Supplementary Figure 2: High temperature does not affect the stability of mature miR-451 in erythrocytes. Fresh and boiled blood from **(A)** mice, **(B)** chickens, and **(C)** pigs were used to extract total RNA. RNA samples were then subjected to quantitative analysis of miR-451 level by qRT-PCR. The Y-axis shows C_T value of miR-451 PCR. miR-451 level in *miR-144/451* KO blood was used as negative control. Data were from 3 biological replicates. Note: there is no significant difference of miR-451 levels between cooked and uncooked blood.

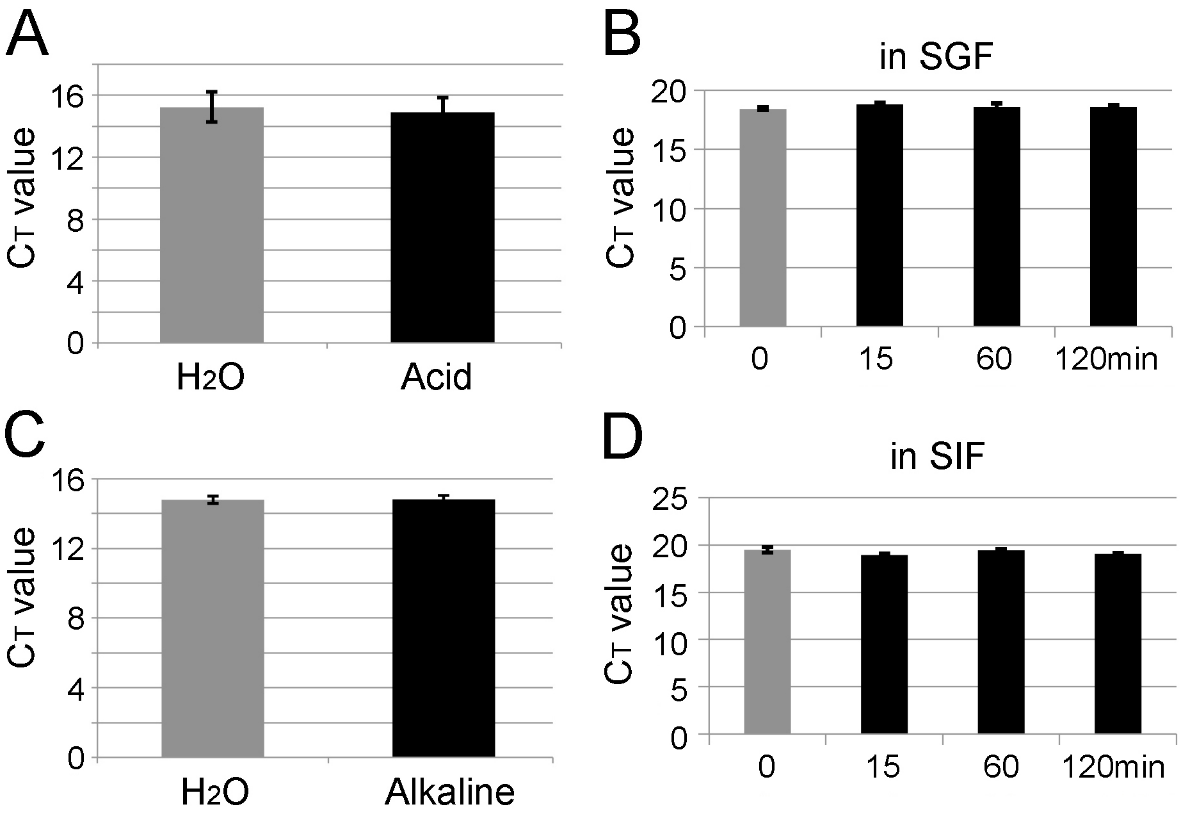

Supplementary Figure 3: Acidification and alkalization do not affect the stability of mature miR-451 in erythrocytes. Blood was acidified by **(A)** HCl to pH 2.5 or **(B)** a simulated gastric fluid (SGF). Total RNA samples isolated from WT blood were **(C)** alkalized by NaOH to pH 8.5 or **(D)** a simulated intestine fluid (SIF). After incubation at 37°C for two hours, all samples were subjected to measurement of miR-451 level by qRT-PCR. The Y-axis shows C_T value of miR-451 PCR (n=5). Note: there are no significant differences of miR-451 level between acid- or alkaline-treated and un-treated blood or RNA samples.

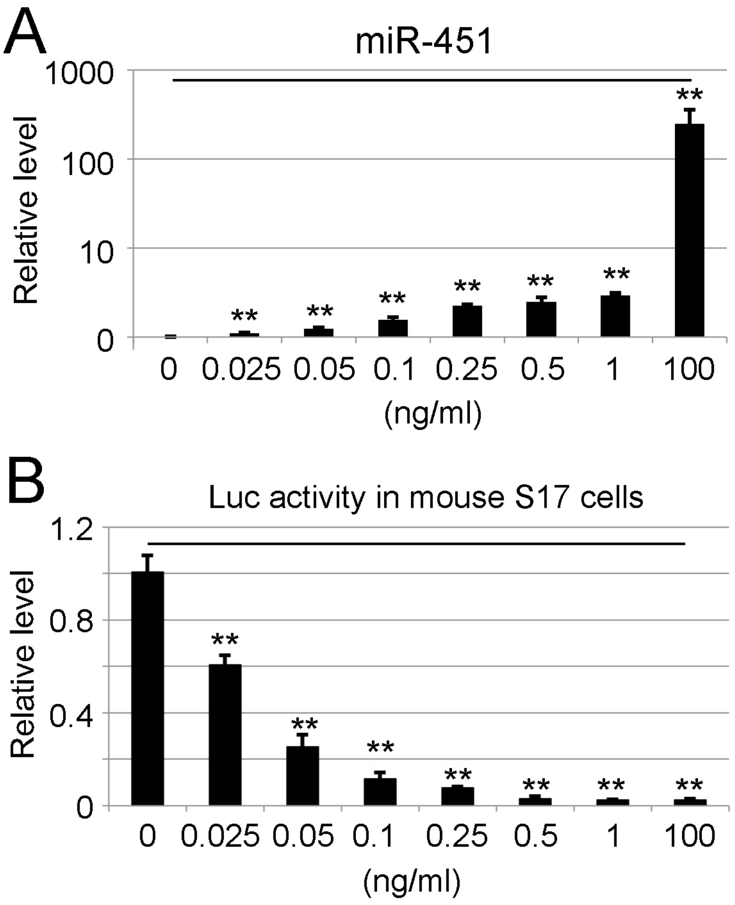

Supplementary Figure 4: Trace amount of miR-451 sufficiently inhibits Ywhaz mRNA. (A) Fold change of miR-451 level in S17 stromal cells derived from mouse bone marrow after transfection of miR-451 retroviral vector. Relative expression levels of miR-451 are shown on the Y-axis (log scale). X-axis shows the concentration of expression vector transfected to S17 cells. n=3 wells of S17 cells. **p-value < 0.01 (*t*-test). **(B)** Interaction between miR-451 and the Ywhaz 3'UTR inhibits expression of a linked luciferase reporter gene. Firefly luciferase cDNA was fused to the normal 3'UTR of Ywhaz cDNA. The reporter constructs were cloned into pGL3-BS expression vector and transfected into S17 cells along with the miR-451 expression construct and a constitutively active Renilla luciferase control plasmid. Luciferase activities were determined 24 hours post-transfection. The Y-axis indicates firefly/renilla luciferase activity; with levels from reporter vector lacking transfection of miR-451 vector assigned an arbitrary value of 1. Results indicate the average of three separate experiments. **p < 0.01 (*t*-test).
